# *α*-Mangostin Alleviated Lipopolysaccharide Induced Acute Lung Injury in Rats by Suppressing NAMPT/NAD Controlled Inflammatory Reactions

**DOI:** 10.1155/2018/5470187

**Published:** 2018-10-10

**Authors:** Mengqing Tao, Jia Jiang, Lin Wang, Yan Li, Qingcheng Mao, Jiyang Dong, Jian Zuo

**Affiliations:** ^1^Yijishan Hospital of Wannan Medical College, Wuhu 241000, China; ^2^Department of Pharmaceutics, School of Pharmacy, University of Washington, Seattle, Washington 98195, USA; ^3^Department of Electronic Science, Xiamen University, Xiamen 361005, China

## Abstract

*α*-Mangostin (MAN) is a bioactive xanthone isolated from mangosteen. This study was designed to investigate its therapeutic effects on acute lung injury (ALI) and explore the underlying mechanisms of action. Rats from treatment groups were subject to oral administration of MAN for 3 consecutive days beforehand, and then ALI was induced in all the rats except for normal controls via an intraperitoneal injection with lipopolysaccharide. The severity of disease was evaluated by histological examination and hematological analysis. Protein expressions in tissues and cells were examined with immunohistochemical and immunoblotting methods, respectively. The levels of cytokines and nicotinamide adenine dinucleotide (NAD) were determined using ELISA and colorimetric kits, respectively. It was found that MAN treatment significantly improved histological conditions, reduced leucocytes counts, relieved oxidative stress, and declined TNF-*α* levels in ALI rats. Meanwhile, MAN treatment decreased expressions of nicotinamide phosphoribosyltransferase (NAMPT) and Sirt1 both* in vivo* and* in vitro*, which was accompanied with a synchronized decline of NAD and TNF-*α*. Immunoblotting assay further showed that MAN downregulated HMGB1, TLR4, and p-p65 in RAW 264.7 cells. MAN induced declines of both HMGB1/TLR4/p-p65 and TNF-*α* were substantially reversed by cotreatment with nicotinamide mononucleotide or NAD. These results suggest that downregulation of NAMPT/NAD by MAN treatments contributes to the alleviation of TLR4/NF-*κ*B-mediated inflammations in macrophage, which is essential for amelioration of ALI in rats.

## 1. Introduction

Xanthone is a naturally occurring polyphenol with the hybrid structure of flavone and anthraquinone. Similarly, xanthones also possess various notable bioactivities and have drawn intense attentions worldwide [1]. Increasing researches are conducted to synthesize more effective xanthone derivatives and to explore their possible clinical applications [1, 2]. Among the well investigated bioactivities, the most eye-catching finding is the encouraging potential in treatments of inflammatory diseases [1–3].

Although numerous studies have solidly confirmed the anti-inflammatory properties of xanthones both* in vivo* and* in vitro*, the underlying mechanisms are still not well understood. It is believed that, as a typical polyphenol, the beneficial effects of xanthone on inflammations result from its antioxidative activities, and downregulation of certain oxidative stress sensitive pathways such as COX-2, NF-*κ*B, and MAPKs is responsible for reduced inflammatory reactions [4–8]. Indeed, these molecular events are essential for the alleviation of inflammatory manifestations, as all of them are deeply implicated in the development and progression of inflammatory diseases. Consistent with these findings, we also reported that xanthone derivatives substantially inhibited the activation of NF-*κ*B in rodent arthritis models, which subsequently alleviated the arthritis severity [9–11]. However, the pathways discussed above just serve as part of downstream in the inflammatory cascade, and identification of the molecular targets of xanthones contributing to these critical signaling changes is important for better understanding of their anti-inflammatory mechanisms.

Recently, we carried out a metabolomics study to elucidate therapeutic mechanisms of *α*-mangostin (MAN, a mangosteen derived prenylated xanthone) on experimental arthritis and found that MAN compromised the biosynthesis of nicotinamide adenine dinucleotide (NAD) in fibroblast-like synoviocyte (FLS) via downregulation of nicotinamide phosphoribosyltransferase (NAMPT, a rate-limiting enzyme in the salvage pathway) and subsequently protected the joints from destruction (unpublished). We believe that the decrease in energy metabolism during this process is critical for the clinical outcome. However, the effects of MAN in FLS could have little to do with the alleviated systemic symptoms, because most of the circulating extracellular NAMPT (eNAMPT) and NAD are contributed by leucocytes [12, 13]. From this point of view, manipulation of NAMPT/NAD in blood cells by xanthone would be much more meaningful in treatments of systemic inflammatory diseases, and exploration of such knowledge will be beneficial to further understand its anti-inflammatory mechanisms. Therefore, in this study, we investigated the therapeutic effects of MAN on lipopolysaccharide induced acute lung injury (ALI) in rats and analyzed its relevance to the regulation of NAMPT/NAD mediated signaling transductions in leucocytes. The results obtained suggest that MAN treatment significantly downregulates NAMPT/NAD and intervenes in energy metabolism, which largely accounts for the alleviation of TLR4/NF-*κ*B mediated inflammations.

## 2. Materials and Methods

### 2.1. Chemicals and Reagents

MAN with the purity of 98% (based on HPLC-UVD analysis) was brought from SanHerb Bioscience Inc. (Chengdu, Sichuan, China). Lipopolysaccharide (LPS, from the Gram-negative bacteria* E. coli* 055:B5), phosphate buffered saline (PBS), BCA protein quantitative kit, NAD, nicotinamide mononucleotide (NMN), HRP conjugated streptavidin, and HRP/biotin conjugated secondary antibodies were purchased from KeyGen Biotech (Nanjing, Jiangsu, China). Fetal bovine serum (FBS) and Dulbecco's Modified Eagle Medium (DMEM) were from TianHang Biotechnology (Hangzhou, Zhejiang, China). Enhanced chemiluminescence (ECL) detection kit, defatted milk powder, and bovine serum albumin (BSA) were from Thermo Scientific (Rockford, IL, USA). Primary antibodies used in both western blot and immunohistochemical assays were obtained from Affinity Biosciences (Cincinnati, OH, USA). Ultrapure water was prepared by using a Milli-Q purification system (Millipore, Bedford, MA, USA).

### 2.2. LPS Challenge in Rats and Treatments

Male SD rats (about 160 g in weight, obtained from Qinglongshan Laboratory Animal Company, Nanjing, Jiangsu, China) were used in this study. All the animal protocols described below were approved by the Ethical Committee of Yijishan Hospital, Wannan Medical College (No. YJS 2018-5-011), and were strictly in accordance with the Guide for the Care and Use of Laboratory Animals (US National Research Council, 2011). Before experimental procedures, all the animals were kept for 7 days to get accommodated. Afterwards, the rats were assigned into 4 groups randomly. Two MAN treated groups received MAN treatments by gavage (suspended in CMC-Na solution) for 3 consecutive days and 15 and 45 mg/kg/day served as the low and high doses, respectively. The other two groups were used as normal healthy and ALI model controls and treated with 0.5% CMC-Na instead. One hour after the last administration, lung injury was induced in all rats except for the normal controls via an intraperitoneal injection of LPS at the dose of 10 mg/kg. The animals were then intensely observed and sacrificed under anaesthesia with chloral hydrate 12 h later. The whole blood was collected through abdominal aorta into anticoagulation/promoting coagulation tubes. Anticoagulated blood was used for complete blood count (CBC) analysis on an automated hematology system (ADVIA 120, Bayer Diagnostics, German), and the coagulated blood was used to separate serum for the serological analyses. The levels of superoxide dismutase (SOD) and malondialdehyde (MDA) in serum were measured using colorimetric kits (JianCheng Bioengineering Institute, Jiangsu, China), and the concentrations of TNF-*α* and eNAMPT in serum were determined using ELISA kits (R&D Systems, Minneapolis, MN, USA) according to the manufacturer's instructions. The lung was promptly dissected, washed by cold PBS, and fixed in buffered formalin for further examinations.

### 2.3. Histological and Immunohistochemical Examinations

The fixed lung was embedded in paraffin and then sectioned at 4 *μ*m thickness. After the staining of hematoxylin and eosin, the lung injury was evaluated based on the following histological changes: (a) thickness of the alveolar walls, (b) infiltration or aggregation of inflammatory cells, and (c) alveolar hemorrhage [14]. Each pathological change was scored on the scale of 0-3 based on the severity: 0, no visual changes; 1, slight; 2, obvious; 3, severe. The highest score in sum for each rat is 9 theoretically. Some deparaffinized sections were rehydrated in PBS and treated with 3% hydrogen peroxide at room temperature for 20 min. The heat-mediated epitope retrieval method was adopted to recover antigen reactivity using citric acid treatment by the aid of microwave heating. The nonspecific proteins were blocked with normal goat serum. Then, the blocked slides were incubated with anti-NAMPT or Sirt1 rabbit polyclonal antibodies (dilution ratio 1: 100) at 4°C overnight and subsequently subject to incubation with the goat anti-rabbit IgG biotin-labeled secondary antibody (dilution ratio 1: 2000) for 1 h at room temperature. Signals of tagged proteins were amplified and detected by HRP-conjugated streptavidin incubation and 3,3-diaminobenzidine (DAB) staining. Finally, the slides were counterstained with hematoxylin, and observed using an Olympus BH-2 light microscope coupled with a digital camera (Tokyo, Japan). Specificity of the immune reactions was tested by negative and positive controls. Certain lung specimens treated with PBS instead of primary antibodies were adopted as negative control, and synovium tissue from rats joint was used as positive controls.

### 2.4. Cells Culture and NAD Determination

Because macrophage is deeply implicated in sepsis and relevant complications, we used RAW 264.7 cells (Jennio Biotech Co., Ltd., Guangzhou, China) in the following experiments to mimic effects* in vivo*. The cells were grown in DMEM supplemented with 10% FBS and penicillin-streptomycin (100 U/ml) at 37°C under the humid atmosphere containing 5% CO_2_. Cells were passed every 2 days.

To evaluate the effects of MAN on production of NAD, cells were seeded in a 75 cm^2^ culture flask and treated with MAN with a series of concentrations or in combination with NAD/NMN after an overnight incubation for attachment. After 24 h treatment, the cells were collected and counted. NAD was extracted from cells using the reagent provided in the NAD determination kit with the aid of heating in a boiling water bath (Solarbio biotech, Beijing, China), and supernatant of the extraction was obtained after a high speed centrifugation (10,000 g) at 4°C. The concentrations of NAD in the processed samples were determined according to the manufacturer's instructions and then normalized to the number of cells to calculate the relative NAD levels in cells.

### 2.5. Western Blot and ELISA Analysis

Cells at exponential growth stage were seeded into 6-well plates at the density of 1×10^5^ cells/well. According to the experimental arrangement, some cells were pretreated with LPS (1 *μ*g/ml) for 1 h after attachment. Afterwards, the cells were treated with MAN or in combination of NAD/NMN for 24 h based on a predetermined schedule. By the end of treatments, supernatants of the culture medium were collected for TNF-*α* and eNAMPT quantitative analyses using commercial available ELISA kits (R&D Systems, Minneapolis, MN, USA), and the harvested cells were lysed on ice using RIPA buffer supplemented with 1% PMSF. Supernatants from the lysates were obtained after centrifugation at 12,000 RPM under 4°C. Samples containing the equal amount of proteins (10 *μ*g, quantified by BCA method) were subject to SDS-PAGE, and the separated proteins were then transferred onto nitrocellulose filter membranes, which were then blocked with 5% defatted milk for 2 h at room temperature and incubated with primary antibodies at 4°C overnight. The target proteins on the membranes were probed by HRP conjugated secondary antibodies (2 h at room temperature), and specific signals of protein complexes were finally developed and detected using an ECL detection kit on a Tanon 5200 system (Bio-tanon, Shanghai, China) [15].

### 2.6. Molecular Docking Simulation

The 3D conformation construction and energy minimization of MAN were achieved using the ChemBio3D Ultra 14.0 software (Cambridge, MA, USA). Crystal structures of NAMPT in complex with small molecular ligands were chosen and downloaded from RCSB Protein Data Bank. The docking simulation procedures were mainly performed on the 5LX3 conformation, while 3DKJ and some other cocrystallized structures were used to test results. To prepare the protein for simulation procedures, atoms of water and ligands were removed from the molecule, and polar hydrogens were added by the aid of AutoDockTools 1.5, the graphical interface of AutoDock software. MAN and NAMPT structural data were then converted into the PDBQT format and fed to AutoDock 4.2 for docking simulation. A grid box with the size of 40×40×40 was generated to completely encompass the binding site cleft with the coordinate of PHE 193 as the center reference. Lamarckian genetic algorithm (GA) was adopted. Interactions between MAN and the protein were analyzed and visualized using PyMol graphic system 2.1 (DeLano Scientific, San Carlos, CA) [16].

## 3. Results 

### 3.1. MAN Treatment Alleviated ALI Severity in Rats

**Figure 1:**
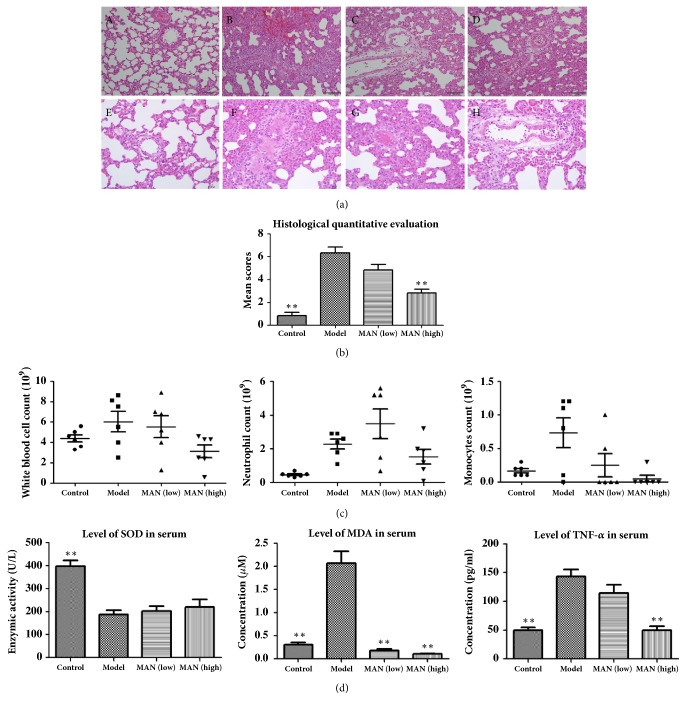
Therapeutic effects of MAN on ALI in rats. (a) Histological examination of lung in rats: A-D (100 × magnification), E-H (400 × magnification), representative images selected from normal control, ALI model, MAN treated (low), and MAN treated (high) groups, respectively; (b) quantitative evaluation of pathological changes based on histological examination; (c) main cell types with significant population differences among groups; (d) serological differences among groups. Statistics significance: ^*∗∗*^*p < 0.01* compared with ALI models.

LPS challenge caused severe acute lung injury in rats. The airspace inflammation was characterized by notable alveolar thickening, interstitial edema, and extensive inflammatory cells infiltration. Interstitial patchy hemorrhage was readily observed too. MAN treated rats showed modest lung injury, but the severity was much less than pathological changes in ALI models. MAN at high dose effectively reduced interalveolar septal thickening and alveolar hemorrhage, and cells infiltration was also relieved (Figure 1(a)), which together resulted in a significant reduction in histological scores of lung in rats (Figure 1(b)). CBC analysis revealed that the most significant hematological change under LPS challenge was the enlarged leucocytes population, supporting the inflammatory conditions* in vivo*. Specifically, neutrophil and monocyte were significantly increased. Individual rat's responses varied substantially to MAN regimen, especially in the low dose group; however, the tendency of dose-dependent decrease of these indicators was quite obvious (Figure 1(c)). SOD activity was significantly suppressed in ALI rats, and MAN treatments only slightly recovered it. Meanwhile, MDA production was greatly elevated in ALI models, which was completely reversed by MAN treatments. Similar effects of MAN treatments occurred to the levels of TNF-*α* in serum. The LPS induced overproduction of TNF-*α* was abrogated by MAN in a dose-dependent manner (Figure 1(d)).

### 3.2. MAN Treatment Downregulated NAMPT and Sirt1 in ALI Rats

**Figure 2:**
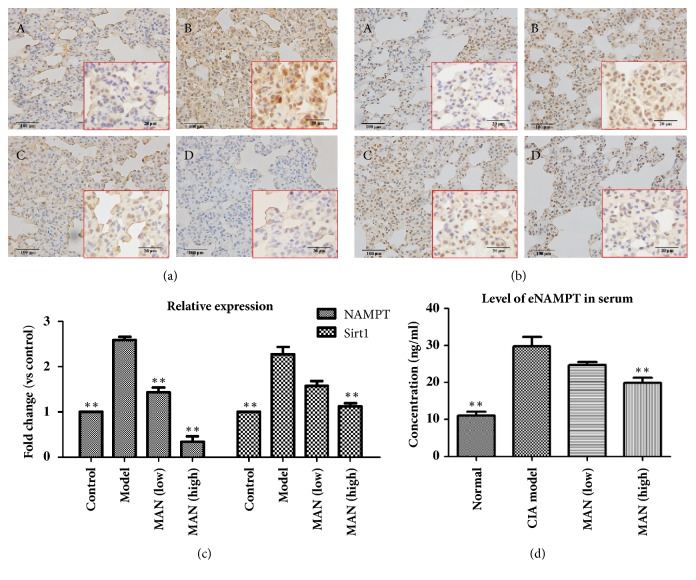
Regulation of MAN on NAMPT and Sirt1* in vivo*. (a) Expression of iNAMPT in lung; (b) expression of Sirt1 in lung; (c) quantitative results of immunohistochemical assays; (d) levels of eNAMPT in serum. Photographs A-D represent normal control, ALI model, MAN treated (low), and MAN treated (high), respectively. Statistical significance: ^*∗∗*^*p < 0.01* compared with ALI models.

The increase in MDA levels in ALI rats suggested that lipid catabolism* in vivo *could possibly be disrupted. This phenomenon reflects the augmented energy expenditure due to extra demands of inflammatory reactions and suggests that the primary energy resource could have been switched to fatty acid oxidation [17]. As well known, NAMPT-Sirt1 axis plays a central role in the maintenance of energy homeostasis in mammals [17]. Such metabolic alteration will inevitably affect the signaling status. Consistent with our assumption, immunohistochemical examination revealed that expressions of both intracellular NAMPT (iNAMPT) and Sirt1 were remarkably enhanced in ALI rats, and these abnormal changes were restored by MAN treatments (Figures 2(a) and 2(b)). Of note, the suppressive effect of MAN on iNAMPT was especially effective. It brought iNAMPT expression in lung down to the extent even lower than that in normal animals (Figure 2(c)). Similar changes happened to eNAMPT in serum. The levels of circulating eNAMPT seem to remain synchronized with its intracellular counterpart, but its fluctuation was a bit smaller (Figure 2(d)). Simultaneous downregulation of iNAMPT and eNAMPT suggested that MAN substantially intervened in the energy metabolism and would eventually affect NAD consuming signaling transductions, including Sirt1.

### 3.3. MAN Downregulated NAMPT and Sirt1 in RAW 264.7 Cells* In Vitro*

**Figure 3:**
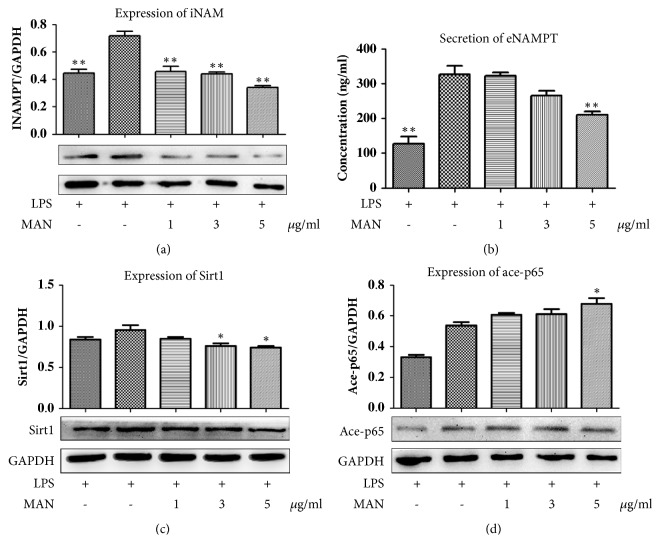
Regulation of MAN on NAMPT and Sirt1 in RAW 264.7 cells* in vitro*. (a) Expression of iNAMPT in cells; (b) levels of eNAMPT in culture medium; (c) expression of Sirt1 in cells; (d) deacetylation capability of Sirt1 indicated by expression of ace-p65 in cells. Statistical significance: ^*∗*^*p < 0.05 *and ^*∗∗*^*p < 0.01* compared with LPS treated cells.

Since leucocytes including macrophage play a critical role in systemic inflammations, we subsequently investigated the effects of MAN on the levels of NAMPT and Sirt1 in RAW 264.7 cells. To mimic the inflammation conditions, the cells were pretreated with LPS, which led to increased productions of iNAMPT, eNAMPT, and Sirt1 (Figures 3(a)–3(c)). Overall, the effects of MAN on these LPS induced changes* in vitro *were similar to those* in vivo*. MAN stimulus reduced both iNAMPT and eNAMPT in a concentration-dependent manner (Figures 3(a) and 3(b)) and exerted similar but smaller effects on the levels of Sirt1 (Figure 3(c)).

Both results from* in vivo* and* in vitro* assays revealed downregulation of Sirt1 by MAN, which is contradictory to a previous report [8]. MTT assay showed that the cells were sensitive to concentration and treatment duration of MAN. By increased exposure to MAN, the viability of cells was dramatically decreased. In the meantime, downregulation of NAMPT/Sirt1 by MAN was diminished and eventually turned to the opposite. These findings suggested that the stress response of the cells under critical conditions should account for the contrary outcomes.

It also raised a concern about the possible negative effects on inflammations brought by reduced Sirt1, since Sirt1 can inhibit the transcriptional activity of NF-*κ*B via deacetylation of p65 subunit [18]. Indeed, we noticed accumulation of ace-p65 upon MAN treatments, which indicated the crippled deacetylation capability of Sirt1 (Figure 3(d)). At present, it is difficult to conclude if such changes will aggravate the inflammation reactions based on limited experimental evidence of this study; however, at least downregulation of Sirt1 by MAN does not seem to make sense to the eased flame. We therefore mainly focused on the changes of NAMPT/NAD in the subsequent experiments.

### 3.4. MAN Inhibits Enzymic Activity of NAMPT by Binding to the Catalytic Site

**Figure 4:**
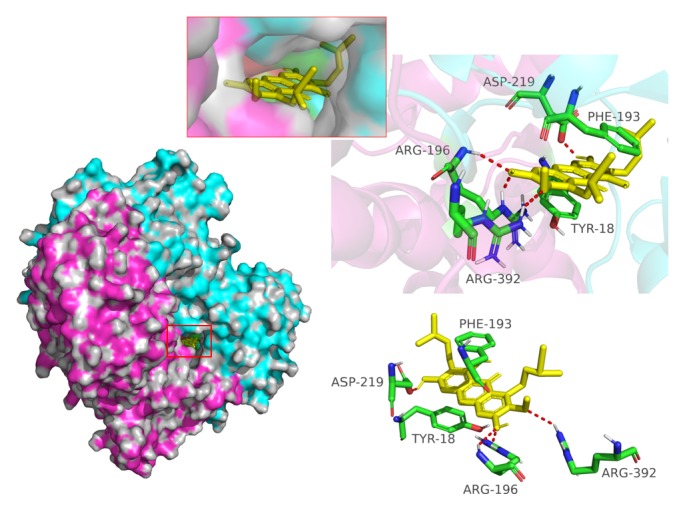
Direct interaction between MAN and NAMPT revealed by molecular docking simulation analysis.

Previously, we found that MAN treatment efficiently reduced production of NAD* in vivo *(unpublished). Given the newly developed conception about feedback between energy metabolism and inflammation [19], manipulation of NAD production by MAN could be crucial for its anti-inflammatory bioactivities. Due to structure similarities with endogenous substrates, we assumed that, apart from its effects on NAMPT expression, MAN could also inhibit the catalytic activity of NAMPT directly. Results generated from simulation docking were clustered and ranked by binding energy, and the one with lowest binding energy was deemed as the most stable conformation and further analyzed. We showed that the energetic gap from the largest conformation population of MAN docking to NAMPT spanned from 7.2 to 8.02 kcal/mol. This docking result provided us a tool to roughly evaluate the stability of constructed complexes and suggested that MAN could bind to NAMPT steadily. As shown in Figure 4, the cleft in active binding site of NAMPT is composed by both A and B subunits of the homodimer (colored by blue and purple, respectively), and the cavity shape was perfectly fit to the structure of MAN. The two residues situated deeply in the cleft (Phe193 from subunit A and Typ18 from subunit B) play critical roles for binding of NAMPT to the ligands [20]. Similar to the interactions with validated ligands, Phe193 and Typ18 formed a strong face-to-face *π*-*π* interaction with the aromatic ring of MAN, and the planar structure of MAN was especially favored for the sandwich-like insertion [20]. Additionally, multiple hydrogen bonds were found between MAN and Asp219 (subunit A), Arg196 (subunit A), and Arg392 (subunit B) in NAMPT, which further stabilized the interactions. As the groove constructed by Phe193 and Typ18 is crucial for substrate binding and Asp219 determines catalytic specificity [20], the occupation by MAN in the binding site could result in reduced catalytic activity of NAMPT.

### 3.5. MAN Inhibited TLR4/NF-*κ*B Mediated Inflammation by Affecting NAD Production

**Figure 5:**
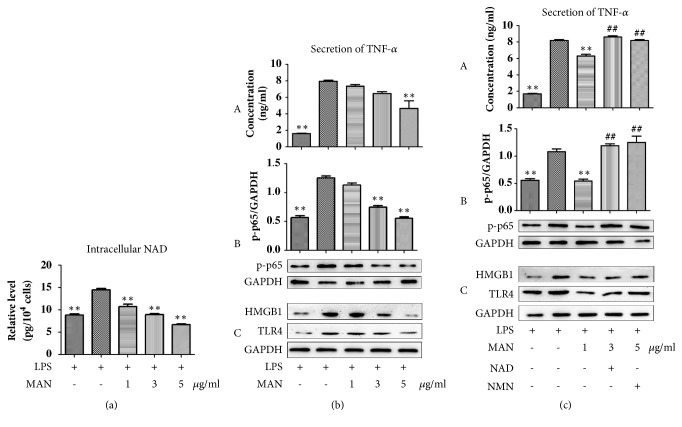
Downregulation of MAN on NAD production contributed to reduce TLR4/NF-*κ*B activation in RAW 264.7 cells* in vitro*. (a) Production of NAD in cells; (b) MAN inhibited activation of TLR4/NF-*κ*B in a concentration-dependent manner; (c) cotreatments with NAD/NMN reversed MAN induced inhibition on TLR4/NF-*κ*B: A, secretion of TNF-*α* by cells; B, expression of p-p65 in cells; C, expression of HMGB1 and TLR4 in cells. Statistical significance: ^*∗∗*^*p < 0.01* compared with LPS treated cells; ^##^*p < 0.01* compared with LPS+MAN treated cells.

Due to the effects of MAN on expression and catalytic activity of NAMPT, biosynthesis of NAD is expected to be suppressed under MAN treatments. As expected, MAN significantly decreased the NAD production in LPS treated RAW 264.7 cells (Figure 5(a)). With the decrease of NAD production, we found a synchronized decline of TNF-*α* in culture medium (Figure 5(b)). Because inflammatory cytokine secretion is universally controlled by the NF-*κ*B pathway, this change would be attributable to the downregulation of NF-*κ*B. This assumption was subsequently proved, as LPS induced expression of p-p65 was completely abrogated by MAN (Figure 5(b)). Meanwhile, MAN treatments reduced the expression of TLR4 and HMGB, an endogenous agonist of TLR4, and partner molecule that amplifies LPS induced TLR4 action [21] (Figure 5(b)). These results suggested that the metabolic changes observed in this study have a logical correlation with TLR4/NF-*κ*B mediated inflammation under MAN treatments.

To further validate the connection between NAD production and TLR4/NF-*κ*B activation, we cotreated the cell with NAD/NMN (main precursor of NAD in mammals) to restore the decreased intracellular levels of NAD under MAN treatments. Cotreatment with both NAD and NMN significantly reversed the MAN induced inhibition of NF-*κ*B activation and TNF-*α* release (Figure 5(c)), which strongly supported our hypothesis. Further, we found that the MAN induced reduction in expressions of TLR4 and HMGB1 was also restored by the supplementary with NAD and NMN (Figure 5(c)). All these evidences suggested that MAN reduced the NAD production via multiple means, which consequently resulted in downregulation of the TLR4/NF-*κ*B pathway and alleviated inflammation.

## 4. Discussion

Due to its high abundance in nature and versatile bioactivities, MAN is one of the most investigated naturally occurring xanthones. Its potentials in therapies of immune and inflammatory disorders are especially meaningful, because MAN rich mangosteen pericarp has been used as a traditional medicine to cure infections and inflammations for centuries in Southern Asia [22]. In previous studies, we noticed that MAN treatments were always accompanied with body weight loss in rats [10] and assumed this could be due to disrupted metabolism* in vivo*. The current study firmly confirmed this hypothesis and further revealed its relevance to the anti-inflammatory properties.

Most researches on anti-inflammatory activities of xanthones mainly emphasized their antioxidative capability [5–7]. We also noticed that the SOD activity could be restored by MAN treatments; however, this effect was not so significant. In comparison, much bigger differences were observed concerning the levels of MDA among groups. Such changes served as an important indicator for relieved oxidative stress and inflammation but could also reflect the altered lipid metabolism profile* in vivo*, because the lipid peroxidation is usually connected to accumulation of fatty acids in blood [23].

Available evidences show that Sirtuins regulate key aspects of lipid metabolism, and Sirt1 promotes lipid mobilization and oxidation of fatty acids [24]. We found MAN downregulated Sirt1 both* in vivo *and* in vitro*. Given its well recognized anti-inflammatory role [25], the reduction in Sirt1 expression seems to deteriorate the inflammatory conditions. However, on the other hand, fatty acid has been recognized as the main energy resource to fuel inflammation reactions [17]. From this perspective, downregulation of Sirt1 would be beneficial to inflammation treatments by reducing energy supply. Nevertheless, at present, we can hardly conclude its net effects and further studies should be performed to resolve this paradox.

Accumulating evidences support that, apart from its decisive role in NAD biosynthesis, extracellular NAMPT also acts as an adipokine cooperating in glucose and lipid metabolism. By exerting an insulin-mimetic effect, NAMPT regulates carbohydrate metabolism and promotes fat deposition. Hence, overexpression of NAMPT is usually linked to development of obesity [13]. Taken the negative effects on body weight into consideration, it is reasonable to assume that MAN would downregulate eNAMPT in serum, and this was confirmed in this study. More importantly, this metabolic change has a profound impact on ALI in rats, as an increasing number of reports suggest that eNAMPT is an emerging proinflammatory cytokine [12, 13] and is implicated in the pathogenesis of ALI [26]. eNAMPT appears to be produced through a posttranslational modification from iNAMPT, although the underlying mechanisms still remains elusive [13]. Yoon et al. found that Sirt1 rigorously controlled eNAMPT secretion via deacetylation of iNAMPT [27]. This clue shed some light on the elucidation of therapeutic effects of MAN on ALI. On one hand, MAN reduced the expression of iNAMPT and inhibited its catalytic activity, which led to decreased biosynthesis of NAD and supply of precursor for eNAMPT production; on the other hand, MAN compromised posttranslational modification of iNAMPT by reducing NAD production and Sirt1 expression. All these factors cooperatively contribute to the declined eNAMPT levels. Taken together, manipulation of NAD production could be essential for the therapeutic effects of MAN on ALI via inhibiting eNAMPT mediated inflammation reactions.

Increased leucocyte counts and neutrophil/macrophage recruitment in lung are important hallmarks of sepsis induced ALI [28], which will contribute to the increase in eNAMPT under pathological conditions [12, 26]. The dramatic reduction in neutrophil/monocyte counts under MAN treatments would benefit the improvement of inflammation in ALI rats by reducing circulating eNAMPT. Moreover, leucocytes are indispensable components in the innate immune system to defend infections based on the identification of pathogen-associated molecular patterns by TLRs. However, hyperactivation of this defensive mechanism will also perpetuate unfavorable inflammations. Results from this study showed that reduction in energy metabolism caused by MAN substantially inhibited TLR4/NF-*κ*B activation in macrophage. Despite we still do not know why the decrease in NAD leads to reduced expression of TLR4, there are some available clues to clarify this phenomenon from other perspectives. Camp et al. have shown that eNAMPT is a unique endogenous agonist of TLR4. It can potently activate the TLR4/NF-*κ*B pathway alone without the need of partner molecules [29]. Logically, downregulation of eNAMPT could result in reduced TLR4/NF-*κ*B activation under MAN treatments. Considering their roles in lipid mobilization and fat deposition, decreases in Sirt1 and eNAMPT under MAN treatments could cut down free saturated fatty acids in blood, which will further curb the activation of TLR4/NF-*κ*B, because they can bind to TLR4 and directly elicit inflammation reactions [30]. Due to the high energy expenditure under inflammation conditions, oxygen supply cannot meet the demands sufficiently, and a hypoxia circumstance develops [17], which would cause necrosis of cells and subsequent release of HMGB1 [31]. These molecular events ultimately strengthen the activation of TLR4 and aggravate the inflammation. MAN treatments achieved a low metabolism status and greatly alleviated hypoxia mediated HMGB1 release. All these evidences suggested that MAN could restore the innate immune intolerance in leucocytes by controlling energy metabolism.

## 5. Conclusion

As a well known naturally occurring bioactive compound, MAN possesses a notable clinical potential in treatments of many diseases. This study provides further evidences to support its anti-inflammatory properties and partially elucidates the underlying mechanisms from a unique perspective. The results of this study suggested that MAN suppressed TLR4/NF-*κ*B mediated inflammation reactions by manipulation of NAMPT/NAD, and the regulation of fat metabolism could be an effective therapeutic strategy in therapies of inflammation related disorders.

## Data Availability

The data used to support the findings of this study are included within the article.
